# Drill for Ausculation

**Published:** 1862-07

**Authors:** Thomas K. Chambers

**Affiliations:** Fellow and Censor of the College of Physicians, Lecturer on Medicine at St, Mary’s School, and Physician to the Hospital


*Drill for Ausculation.* *By* Dr. Thomas K. Chambers, *Fellow and Censor of the College of Physicians, Lecturer on Medicine at St. Mary's School, and Physician to the Hospital.*

I place before you a table where you will find seventeen indications which (as a rule) the combinations of the easily recognized signs of pulmonary auscultation afford. It will be very useful to copy them out several times, that you may have impressed on your minds the exact amount of information which the process is capable of giving. Expect this much and no more, or you will be disappointed. Remember that auscultation tells you nothing of the *nature* of the substance whose morbid presence it has enabled you to detect, The "*fluid in the pleura with solid lung,*" which "*bulged ribs,*" combined with "*dulness*" and "*tubular breathing,*" indicate, may be pus, serum, or blood—the history of the case alone can lead you to know which. The "*cavities*" in solid lung may be tubercular vomicæ, pneumonic abscesses, or dilated bronchi—the ear, unassisted by other records, does not enable you to diagnose. Fine or coarse crackling very often cannot be absolutely determined to be inside or outside the lung—to be the crepitation of the tubes or the rubbing of the pleural surfaces. Do not attempt to decide by auscultation, but by other symptoms.

There are, however, a few by-aids to the diagnosis by auscultation which are worthy of separate mention, in order that you may know how to use them worthily, and to caution you against their abuse.

1. *Whispering Pectoriloquy.*—When the patient whispers a few words of several syllables, and the distinction between the syllables is clearly heard, it is often taken as a sign of a cavity. It is an absolute sign of *diseased lung*, but not an absolute sign of a cavity, unless it is found in a limited spot with a clearly defined line, outside which line the voice is quite different.

While on this subject I will give you a hint about auscultating the voice. Always make the patients speak in words of several syllables; make them count "twenty-one," "twenty-two," &c.; not "one," "two." You get twice as much information thus.

2. *Cracked-pot Sound.*—A jingling noise, like a cracked pot, or more li e the striking of the clasped palms on the knee, as when we amuse children by imitating the jingling of money, is often produced by percussing a superficial large cavity while the patient holds the mouth open. But it is not a sign absolute. I used to show you last year in the wards a child with a cavity at one apex, where the cracked-pot sound never occurred, though it was remarkably distinct, almost always in a perfectly healty part of the opposite lung. It is caused exactly in the same way as the imitative money jingle in the close hand—namely, by the air being *jerked* out of a partially open space. When the parietes of the chest are thin, it may thus be produced by the jerk of the air in the trachea or large bronchi. Do not, therefore, set store by it as a sign.

3. *Metalic Tinkling.*—This is a valuable sign of a very large cavity with small bubbles bursting in it. It is like the sound of a small shot or fragment of gravel falling in a thick metal vase. It shows the existence of either a vast vomica, or of air in the pleura, with a communication open to the bronchi.

I will now go through the eighteen simple combinations into which these tabulated signs may enter, and point out what information you get immediately from them, and in what direction you are to seek for more on which to ground your diagnosis.

*Combination* A 1.—The *flattened ribs* show that some morbid state has existed; there has been either inflammation of the parietal pleura, which has thickened it with contracted scars, or a condensation of the lung for a sufficiently long period to leave cicatrices, and to diminish the volume of the organ. But the recovered *resonance* shows that the lung is again at work—that the fight is over, and that corn again grows on the battle-field.

A 2.—Be quite certain that the *bulging of the ribs* is not merely apparent, and that the other side, being flattened from disease, does not make them seem too rounded and prominent by contrast. If they are really *bulged,* and yet resonant, there must be a larger body than natural beneath the point of percussion. And this air must be either in the pleura (pneumo-thorax) or in the lung (emphysema.) If the former, you find a peculiar resonance like that produced by tapping the stomach under the ribs, you find the breath-sounds quite absent, and probably also "metalic tinkling." If the latter, you have breath-sounds, perhaps bronchial breathing, and not unfrequently a peculiar crackling like the crumpling of fine tissue paper. When you hear this "crumpling," it is very distinctive. It arises, I would suggest, from the rubbing of the rugged, bubbly-looking pleura of the lung against the parietal.

B 1, C 1, and C 4.—Solidification of the lung makes it dull, and makes the voice and breathing bronchial or tubular, whether arising from pneumonic acute condensation, or from such a chronic cause as tubercle. But if the cause be chronic, the ribs will be flattened in proportion to its chronicity and the length of time it has endured.

B 2.—*Dullness* on the *bulged part* of the ribs shows that some matter more solid than air is bulging them. In the lower part of the chest it is most probably fluid, and then you must try, if you can, a confirmation of your diagnosis by making it move about as the patient changes his position from one side to another. In the upper part it may be an aneurism of a trunk vessel, and then you get pain on pressure of the corroded ribs in the place where they are gnawn away by the lump inside. To the diagnosis of a pectoral malignant tumor you can arrive only *par la voie d'exclusion;* but in all the cases I have seen, the veins, by their remarkable prominence in the immediate neighborhood, have led me to the suspicion.

C 2.—The only case where bulging of the ribs is likely to be joined with tubular breathing is where a lung adherent to the ribs is condensed by the pressure of fluid, and then absolute dullness is of course produced.

C 3.—The larger air-tubes sometimes become stiff and hard from chronic degeneration without any hardening of the lung-tissue, especially in old people, and then you get *resonance* combined with *tubular sounds*. The knowledge of this chronic degeneration is important, as it leads you to expect future degeneration in more important viscera—such as of the lung-tissue, in the form of emphysema; of the kidneys, as Bright's disease; of the heart, as dilatation, &c.

D 1 and E 1.—The flat ribs show that there is chronic disease of some standing, and this will modify the activity of your treatment of the acute action indicated in its first stage by the fine crackling, and in a later stage by the coarse crackling. In such a case as this, the fine crackling is an evidence *against* a vomica; while the coarse crackling (especially if bounded by a sharply-defined line) is the best single evidence you can have of one being already formed.

D 2.—The condition of fine crackling or crumpling as a sign of emphysema has just been mentioned under B 2.

D 3, D 4, and D 5.—When only separate lobules are congested (as in the first stage of pneumonia,) the quantity of air in the lung is not sufficiently diminished for dullness on percussion to be distinguishable by the ear; but when the whole substance of the lung is infiltrated with blood or serum or fibrin, instead of air (as in a more advanced stage,) comparative dullness is to be perceived. However, this is not such absolute dullness as in a further stage, when it is so solidified that no air enters. Then fine crackling ceases, and silence or coarse crackling or tubular sounds take its place.—Then, as the lung again becomes pervious in the course of cure, fine crackling returns; the acoustic condition of *becoming* solid and of *becoming* pervious being the same, though the fluid which causes that condition is different.

E 1, 2, 3, 4, 5.—A *coarse crackling* is distinctive of nothing but the immediate cause of the sound, viz: a thickish fluid in the area where it is found, whether bronchi or vomicæ. It may be associated with all sorts of conditions of lung. You hear sometimes physicians pronounce that there is "bronchitis" in a certain case, and expect those who have consulted them to be satisfied with such a diagnosis, and nothing more. It is no diagnosis at all, and certainly not worth a guinea; for it helps in no degree in the treatment, and gives no information available for prognosis. Every patient with emphysema, chronic or acute catarrh, hard tubercle, vomicæ, pleurisy and what not, has from time to time a secretion in the bronchi (or bronchitis,) and to declare with grave sapience that "there is bronchitis," is simply to put into bad Latin what the patient has been telling *you*, namely: that he has got a cough. No; when you do take your patients to a physician, get something more out of him than that.—*London Lancet—Braithwaite's Retrospect.*

